# Supplementary material for: On demand synthesis of hollow fullerene nanostructures
Source: Nat Commun. 2019 Apr 4;10:1548. doi: 10.1038/s41467-019-09545-8 (PMC6449386; doi:10.1038/s41467-019-09545-8)
Supplement: Supplementary file 1 — Supplementary Information [file 41467_2019_9545_MOESM1_ESM.pdf]

Supplementary Information

On Demand Synthesis of Hollow Fullerene Nanostructures

Fei Han, Ruoxu Wang, Yuhua Feng, Shaoyan Wang, Lingmei Liu, Xinghua Li, Yu Han, and Hongyu Chen

This PDF file includes

Supplementary Table 1
Supplementary Figures 1-16

Supplementary Table

Supplementary Table 1. Volume ratio of metal solution and C_{60} solution

	Silver NPs	Fe_3O_4 NPs	Co NPs
$V_{metal}:V_{C60}$	1:200 to 1:500	1:400 to 1:800	1:100 to 1:200

Supplementary Figures

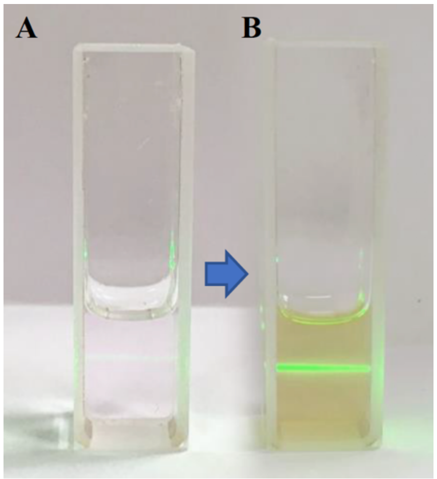

Supplementary Figure 1. Photograph of the nanobowl solution. (**A**) At the very beginning of the reaction. (**B**) After reacted for 36 h.

Note: After the reaction, there was no precipitate at the bottom, all the particles floated in solution. Figure S1A and B were pieced together.

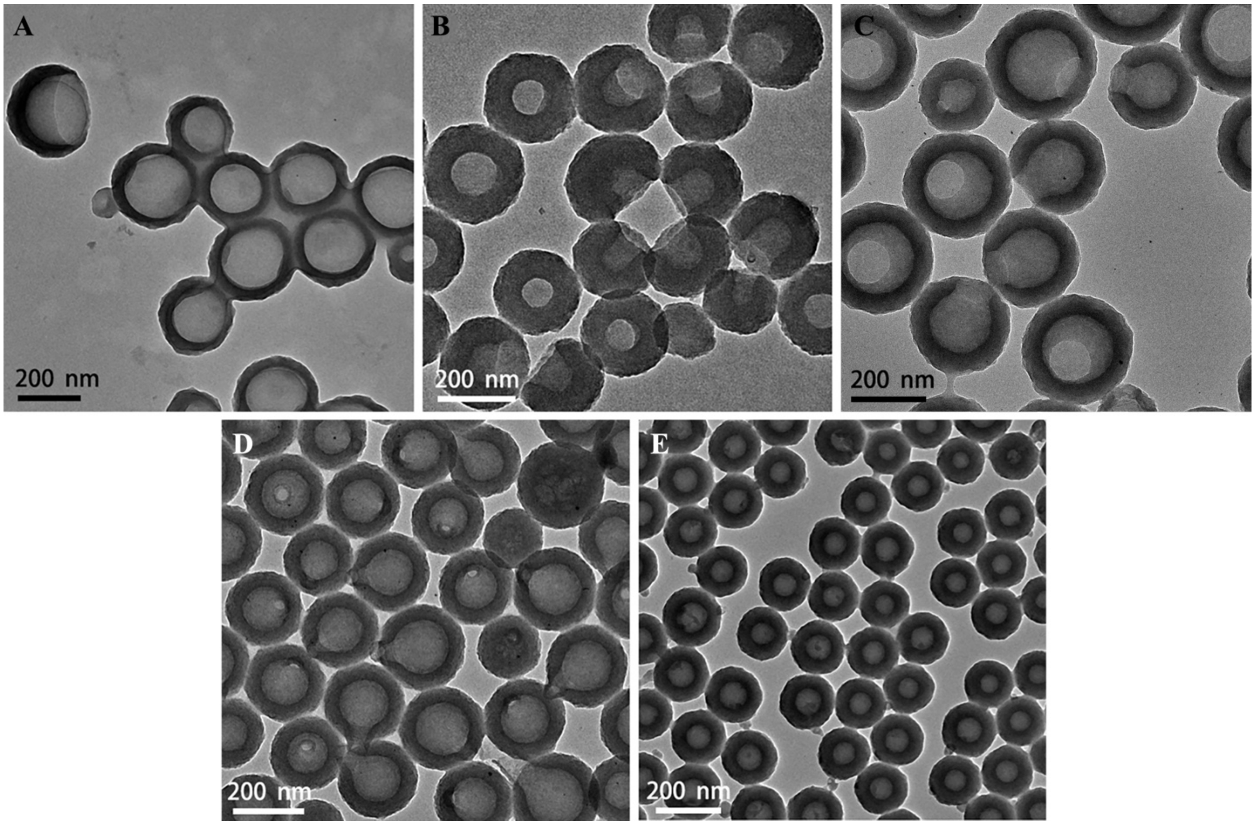

Supplementary Figure 2. TEM images of large area C_{60} hollow structures. (**A-C**) Nanobowls with average opening around 180, 100, and 80 nm, respectively. (**D**) Nanobottles reacted for 24 h, with short bottlenecks. (**E**) Hollow spheres.

Note: With the decreasing of opening size, from A to E, their average external diameter decreased from 265 to 260, 228, 215, and 160 nm, respectively.

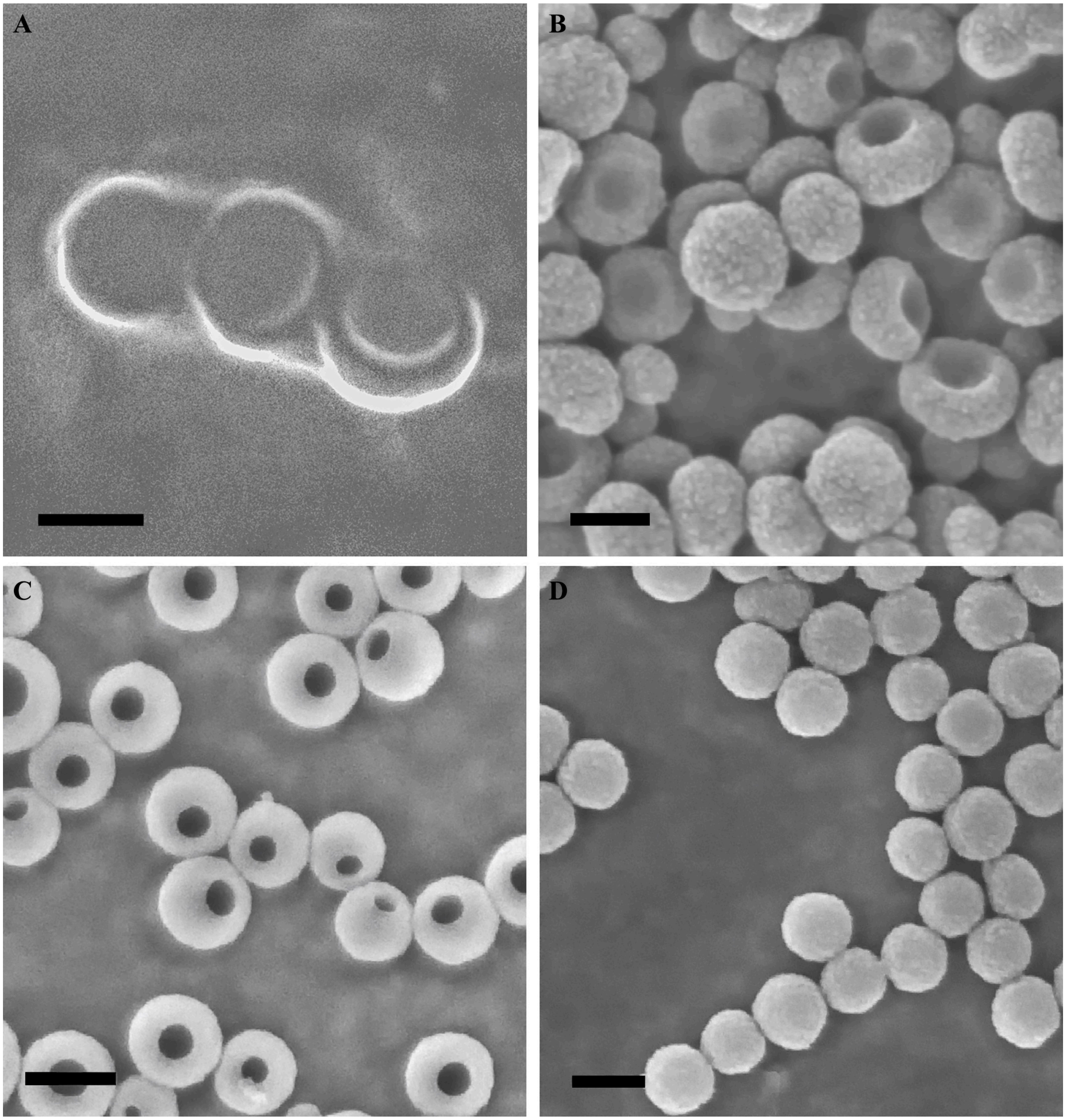

Supplementary Figure 3. SEM images of C_{60} hollow structures with different opening sizes. (**A-C**) Nanobowls with average opening around 180, 100, and 80 nm, respectively. (**D**) Hollow spheres. Scale bar=200 nm.

Note: In some of the SEM images, *e.g.*, S3B, the nanobowls have a tough surface with granular substance presented. This should be the gold layer coated on the sample before SEM analysis.

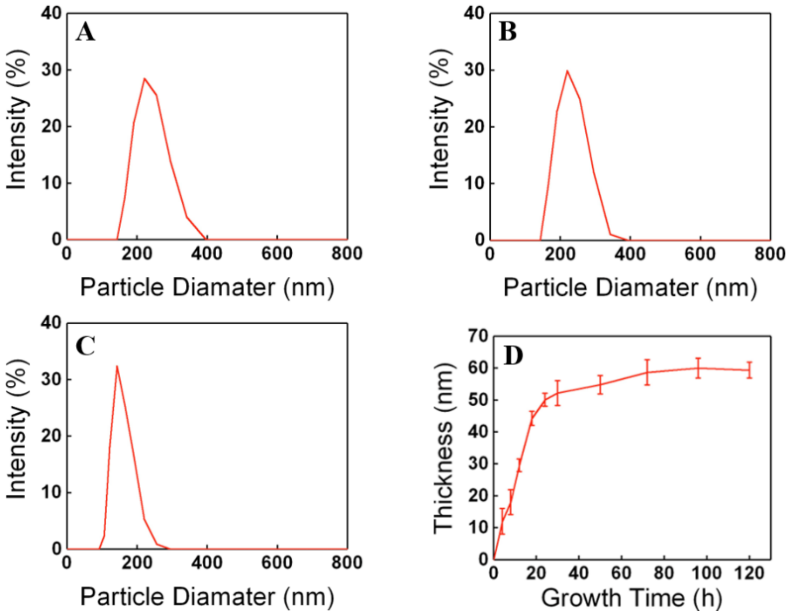

Supplementary Figure 4. DLS measurement of particle size distribution of (**A, B**) nanobowls with average opening around 180 nm and 100 nm, respectively; (**C**) hollow spheres; and (**D**) Dependence of nanobottles' shell thickness on incubation time; error bars indicating standard deviation.

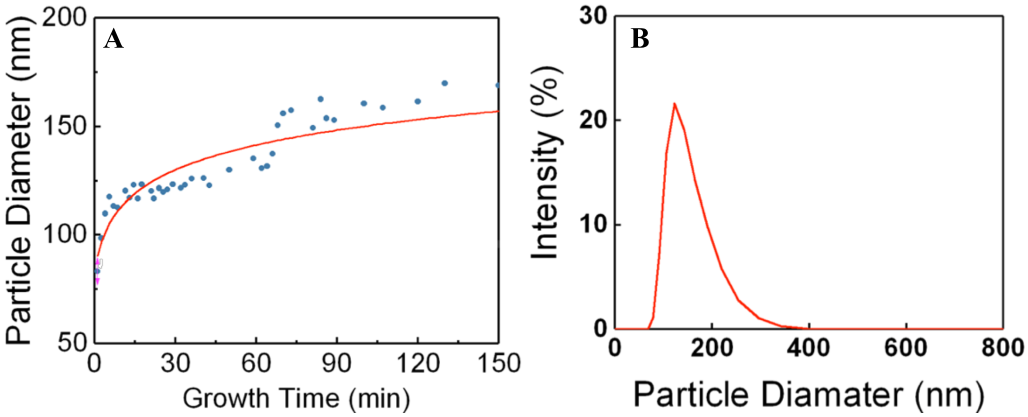

Supplementary Figure 5. (**A**) DLS measurements reflect average particle diameter change in the first 150 min of the reaction. (**B**) DLS measurements of *m*-xylene droplets size distribution in the emulsion of *m*-xylene, DMF, and IPA.

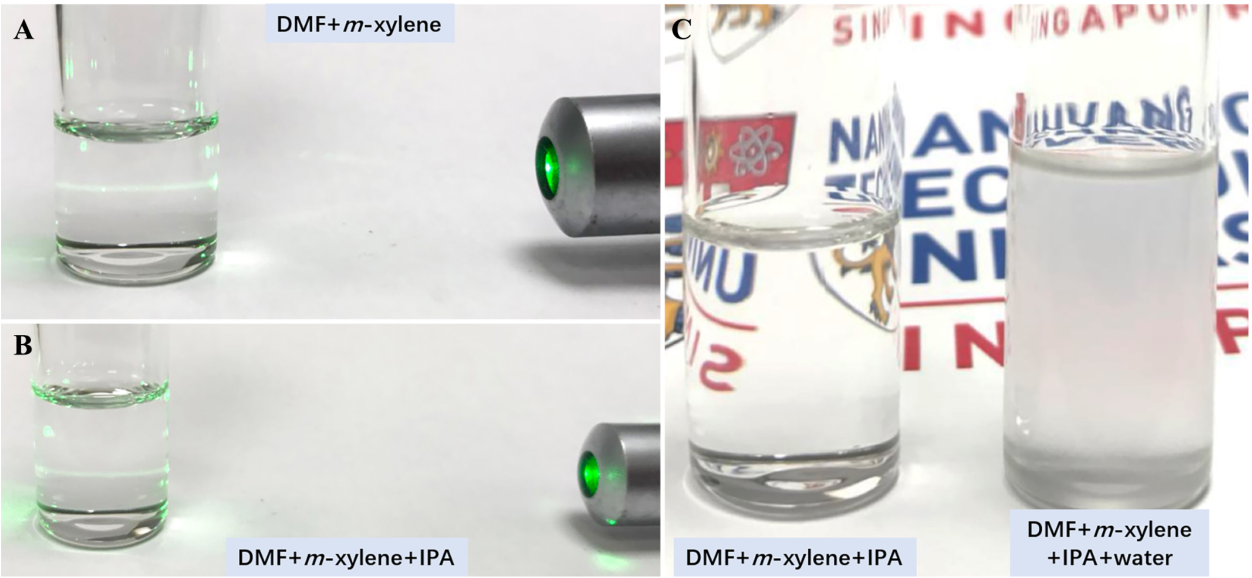

Supplementary Figure 6. Photographs of the mock reaction with the mixture of (**A**) DMF and *m*-xylene; (**B**) DMF, *m*-xylene, and IPA; all mixed according to the ratio in reaction, and (**C**) emulsion obtained after adding 10% volume of water into the above mixture in (B).

Note: For (A) and (B), the mixture appeared to be transparent, but with clear Tyndall effect. We believe that the reason why no emulsion could be observed is because the refractive index of the three components is too close (n= 1.430 for DMF, 1.377 for IPA, and 1.497 for xylene), and *m*-xylene is partly soluble with DMF. For (C), water is miscible with DMF and IPA, but immiscible with *m*-xylene. After adding water, the polarity of DMF-IPA phase was increased, and the phase separation between *m*-xylene and DMF was promoted, and thus, an emulsion was obtained.

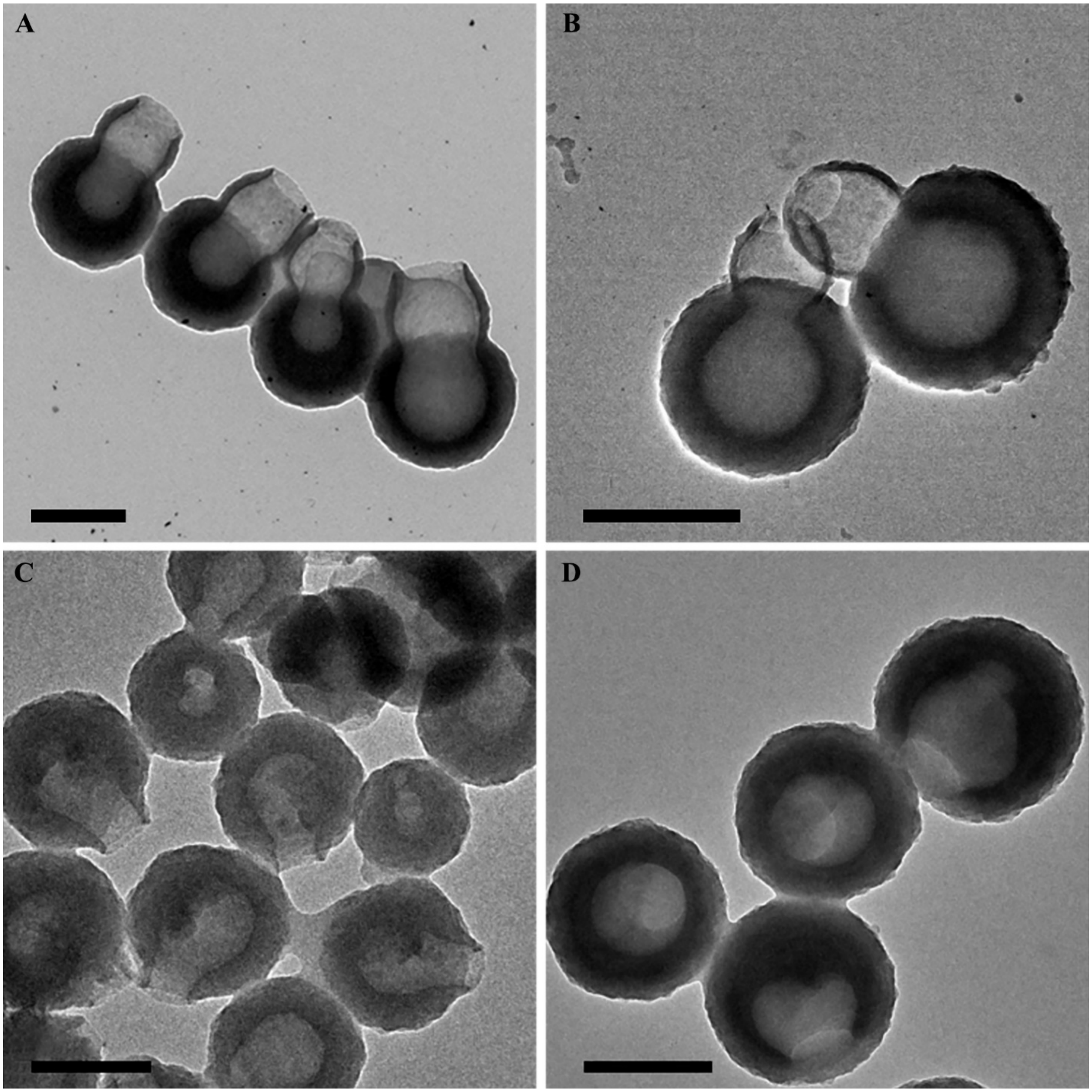

Supplementary Figure 7. TEM images of node growth on C_{60} hollow structures obtained with the volume of C_{60} solution was changed to (**A**) 50 μL, (**B**) 30 μL, (**C**) 15 μL, and (**D**) 3 μL. Scale bar=200 nm.

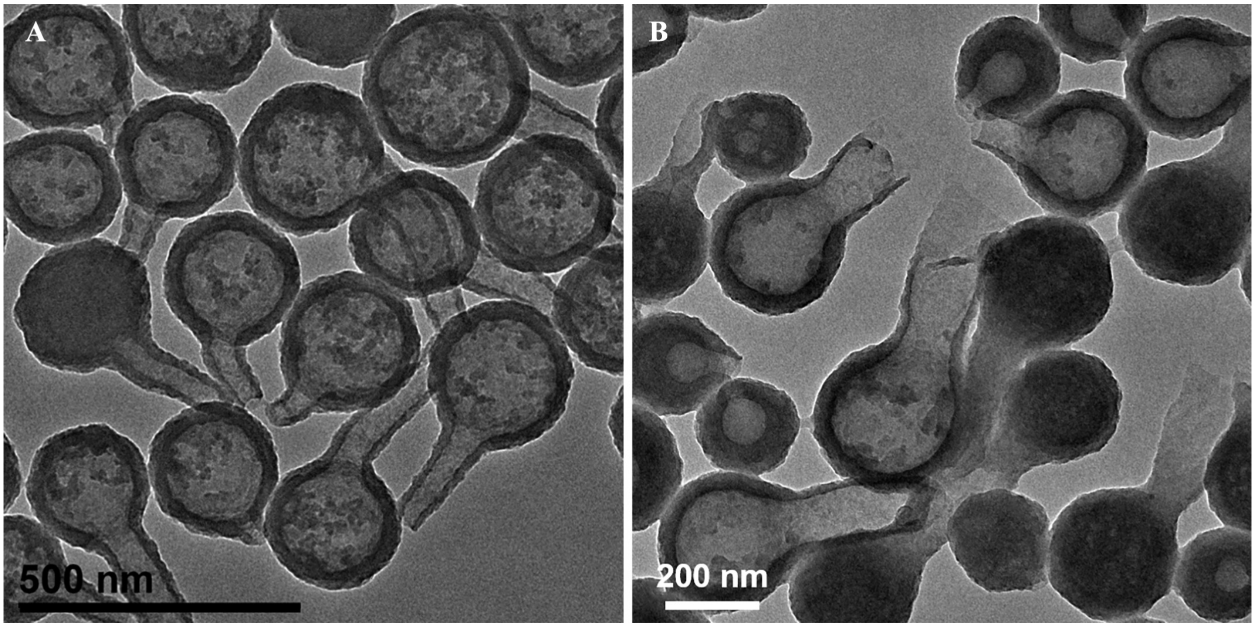

Supplementary Figure 8. TEM image of nanobottles synthesized in water pre-doped DMF (reacted for 36 h). (**A, B**) Contains 1.2%, and 2% volume of water, respectively.

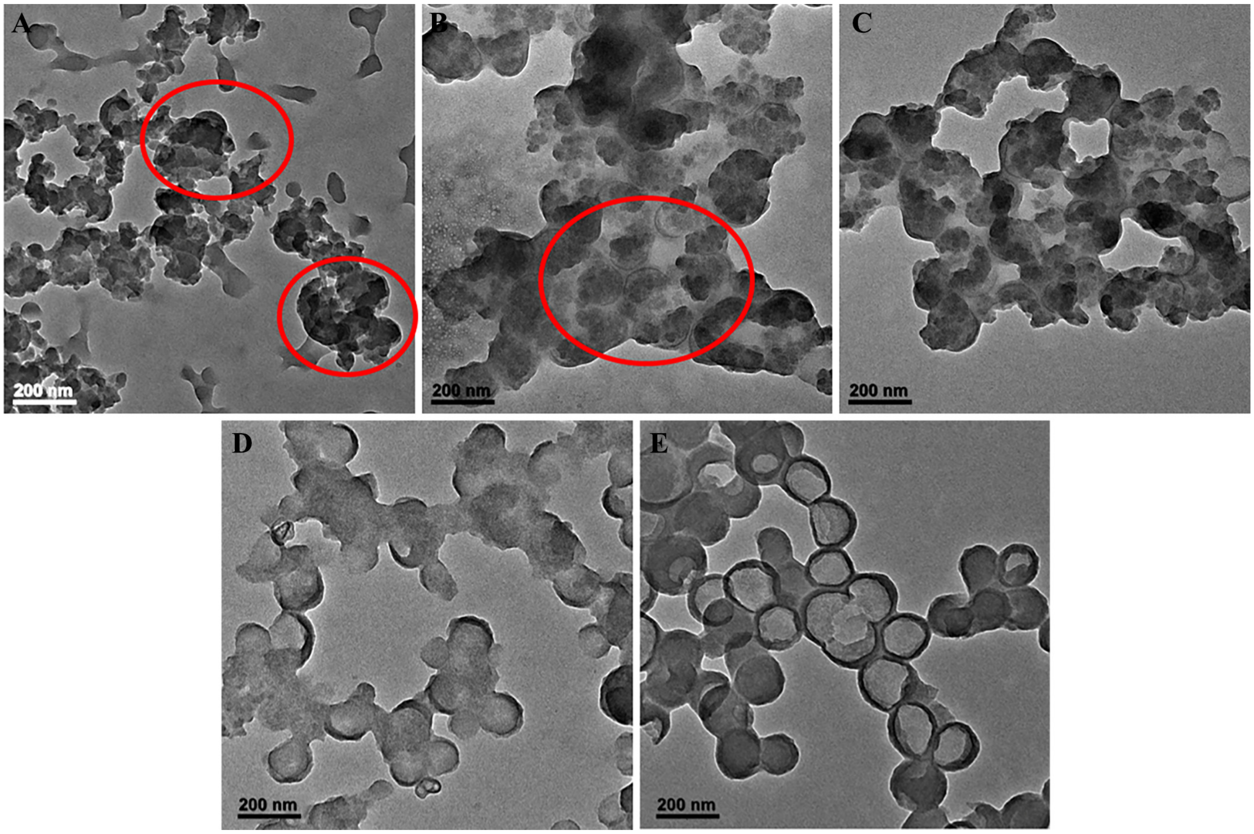

Supplementary Figure 9. (**A-E**) TEM images of C_{60} nanobowl formation intermediates reacted for 1, 2, 3, 5, and 6 h, respectively.

Note: The growth intermediates during the formation of nanobowls showed irregular clusters at 1 h. At 2 h, a very thin spherical shell could be recognized. Within it, there was paste-like substance, which is likely to be the drying pattern of xylene-solvated C_{60}. From 3 h and onwards, the paste-like substance gradually disappeared and the half-shell grew towards complete sphere. We note that no solid template could be observed. From 2-6 h, outer diameter of the half-shell increased from 150 nm to 170 nm, while their inner diameter almost kept unchanged (~140 nm).

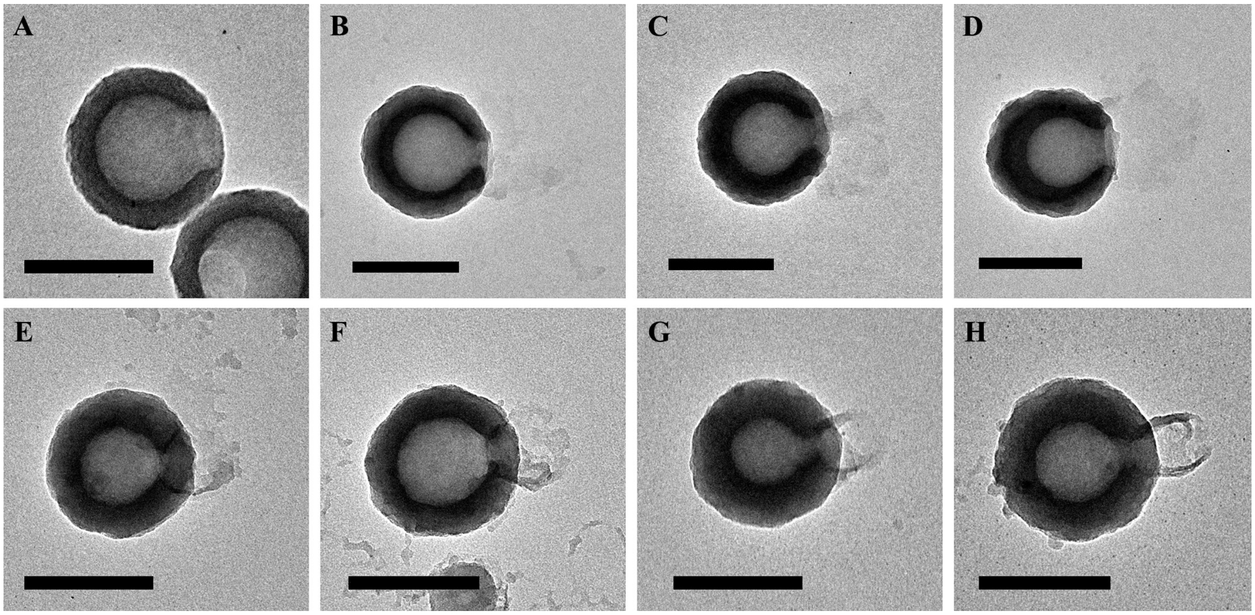

Supplementary Figure 10. (**A-H**) TEM images of C_{60} nanocucurbit formation intermediates reacted for 0.5, 1, 2, 3, 5, 7, 10, and 13 h, respectively. Scale bar=200 nm.

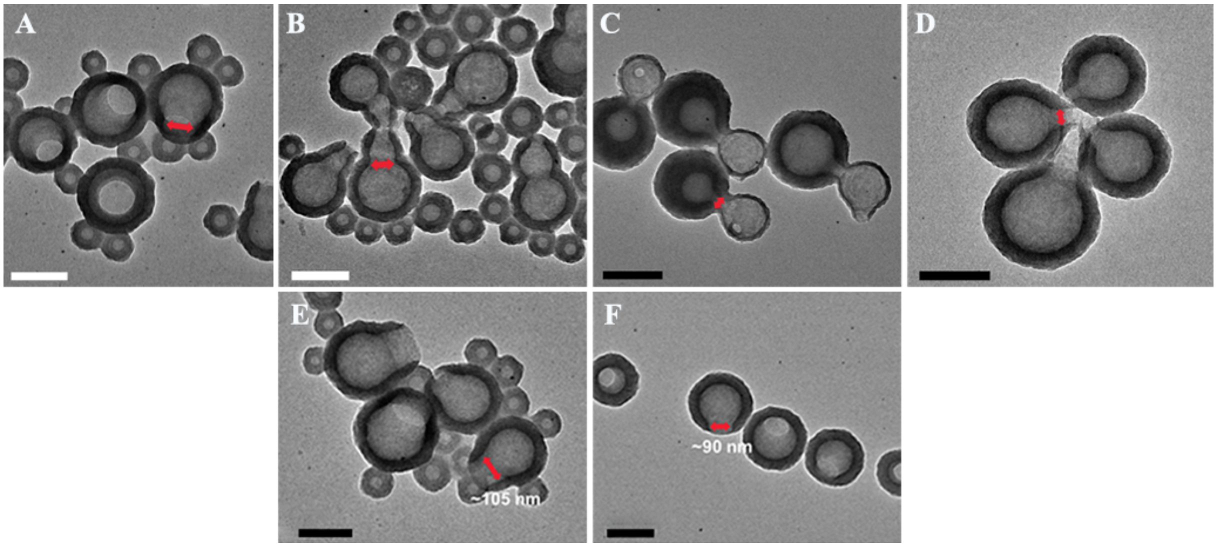

Supplementary Figure 11. TEM images of (**A-C**) cucurbits formed by second addition onto nanobottles reacted for 3, 6, and 15 h, respectively; (**D**) the same nanobottles reacted for 22 h without second addition; (**E**) cucurbits formed by second addition onto nanobowls reacted for 6 h, and (**F**) the same nanobowls reacted for 22 h without second addition. Scale bars are 200 nm.

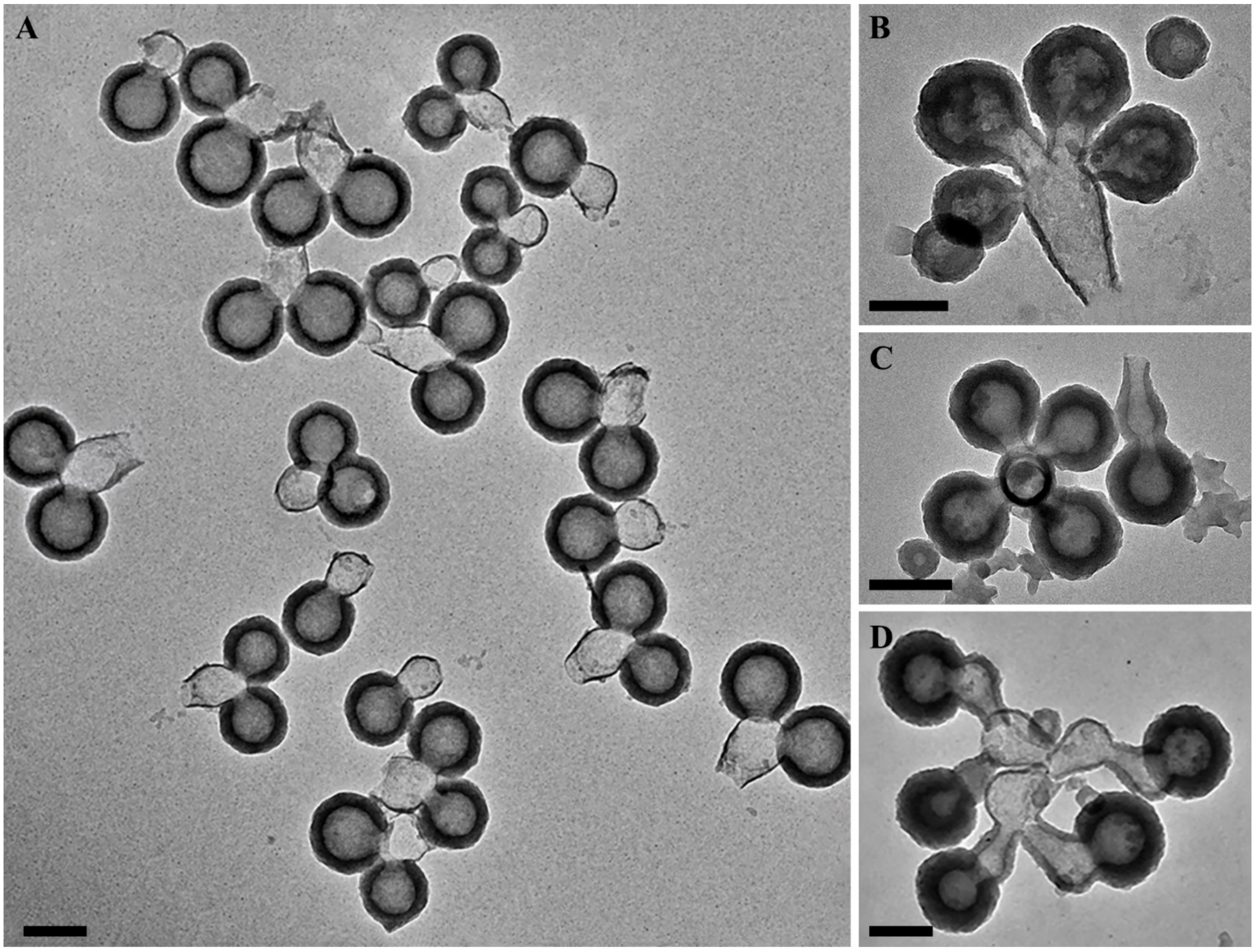

Supplementary Figure 12. TEM image of (**A**) large area dimers of connected nanobowls (contains 1.8% volume of water, with C_{60} volume ratio of the two additions V_1/V_2= 1:0.72); (**B, C**) tetramer of connected bottles with C_{60} volume ratio V_1/V_2= 1:0.72 and 1:0.3; and (**D**) the comparison between connected and non-connected nanocucurbits. Scale bar=200 nm.

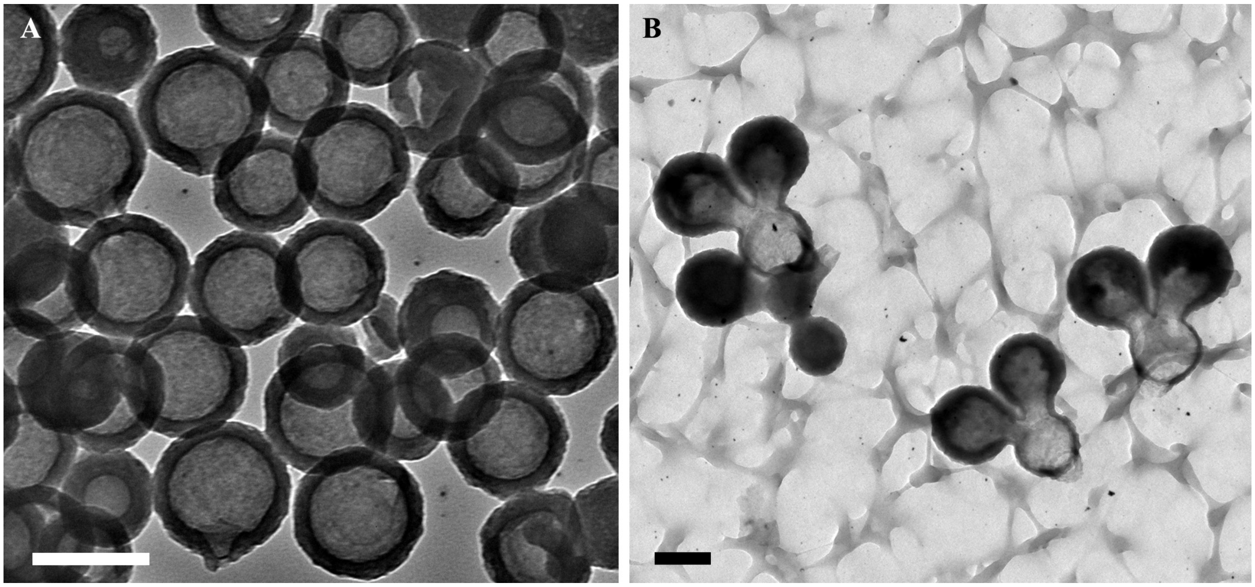

Supplementary Figure 13. TEM image of (**A**) C_{60} nanobottles after vigorously sonicated in water for 1 h, and (**B**) interconnected nanobottles after 3 months storage. Scale bar=200 nm.

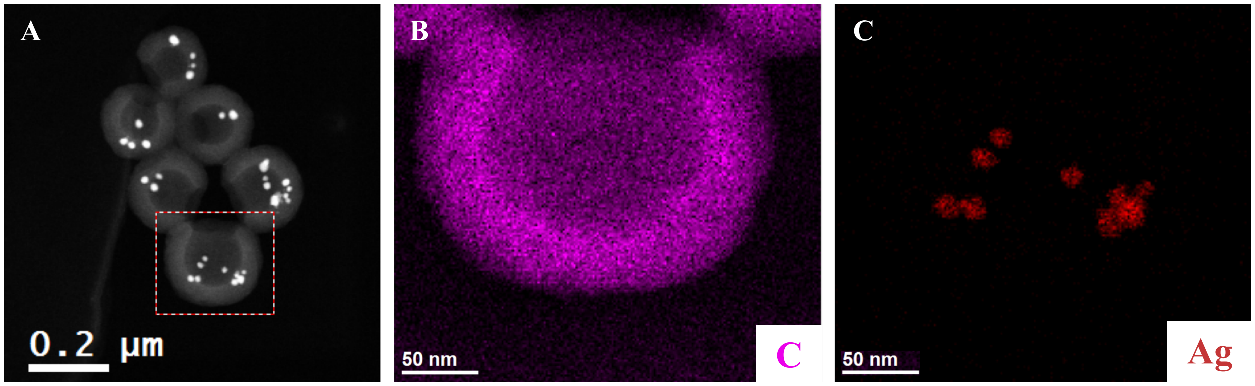

Supplementary Figure 14. (**A**) HAADF image of C_{60} nanobowls holding Ag NPs, (**B, C**) and its elemental mapping of C and Ag, respectively.

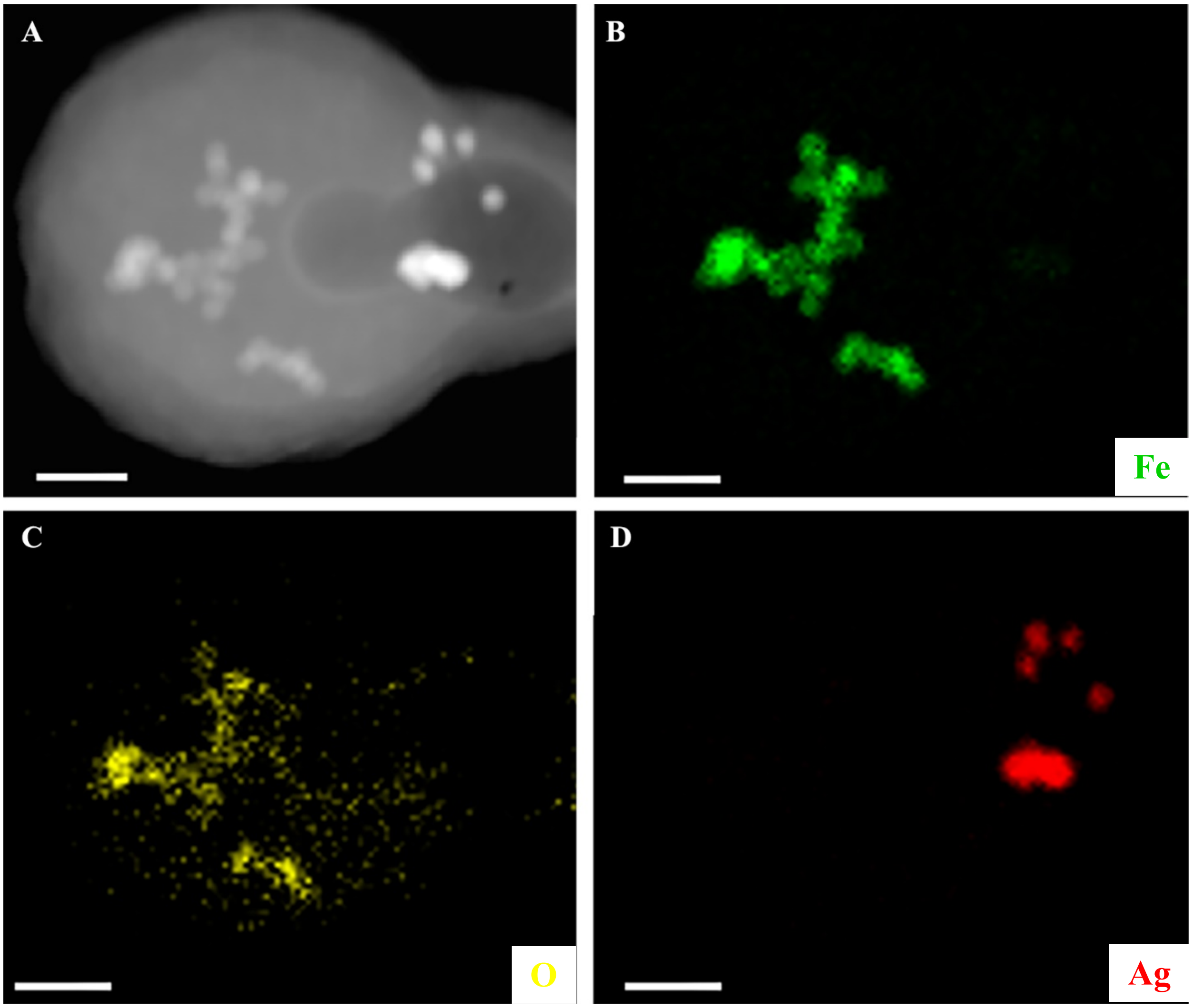

Supplementary Figure 15. (**A**) HAADF image of a typical C_{60} nanocucurbit (the one in Figure 5J and K), with Fe_3O_4 nanoparticles in the bottom node and Ag nanoparticles in the upper node, and (**B-D**) its elemental mapping of C, Fe, and O, respectively. Scale bar=50 nm.

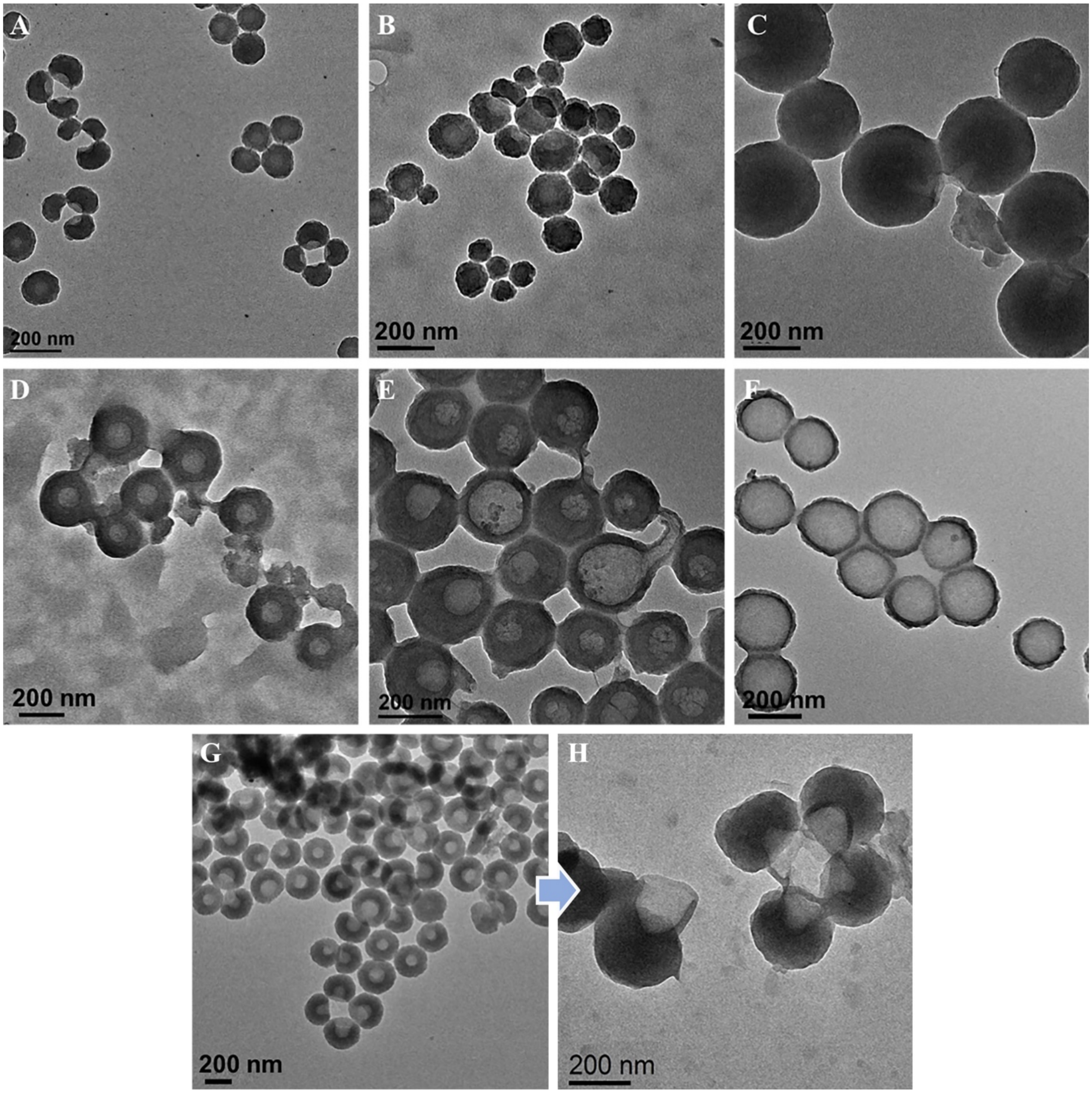

Supplementary Figure 16. TEM images of C_{60} hollow structures obtained with (**A-C**) *o*-xylene, *p*-xylene, and toluene as the good solvent of C_{60}, respectively; (**D**) ethanol as the poor solvent of C_{60}, hollow nano-spheres; (**E**) methanol as the poor solvent of C_{60}, mixture of hollow spheres and nanobottles; (**F**) PCBM nanobowls with very large opening; (**G**) C_{70} nanobowls; (**H**) C_{70} nanocucurbits.
